# Reversible transition between the polar and antipolar phases and its implications for wake-up and fatigue in HfO_2_-based ferroelectric thin film

**DOI:** 10.1038/s41467-022-28236-5

**Published:** 2022-02-03

**Authors:** Yan Cheng, Zhaomeng Gao, Kun Hee Ye, Hyeon Woo Park, Yonghui Zheng, Yunzhe Zheng, Jianfeng Gao, Min Hyuk Park, Jung-Hae Choi, Kan-Hao Xue, Cheol Seong Hwang, Hangbing Lyu

**Affiliations:** 1 Key Laboratory of Polar Materials and Devices (MOE), Department of Electronics, East China Normal University, 500 Dongchuan Road, 200241 Shanghai, China; 2 Key Laboratory of Microelectronics Devices and Integrated Technology, Institute of Microelectronics, Chinese Academy of Sciences, No. 3 Bei-tu-cheng West Road, Chaoyang District, 100029 Beijing, China; 3 University of Chinese Academy of Sciences, 100049 Beijing, China; 4 Department of Materials Science and Engineering, and Inter-University Semiconductor Research Center, College of Engineering, Seoul National University, 08826 Seoul, Republic of Korea; 5 Electronic Materials Research Center, Korea Institute of Science and Technology, 02792 Seoul, Republic of Korea; 6 School of Materials Science and Engineering, College of Engineering, Pusan National University, Busandaehak-ro 63beon-gil 2, Geumjeong-gu, 46241 Busan, Republic of Korea; 7 Wuhan National Laboratory for Optoelectronics, School of Optical and Electronic Information, Huazhong University of Science and Technology, 430074 Wuhan, China

**Keywords:** Electronic devices, Electronic properties and materials

## Abstract

Atomic-resolution Cs-corrected scanning transmission electron microscopy revealed local shifting of two oxygen positions (O_I_ and O_II_) within the unit cells of a ferroelectric (Hf_0.5_Zr_0.5_)O_2_ thin film. A reversible transition between the polar *Pbc*2_1_ and antipolar *Pbca* phases, where the crystal structures of the 180° domain wall of the *Pbc*2_1_ phase and the unit cell structure of the *Pbca* phase were identical, was induced by applying appropriate cycling voltages. The critical field strength that determined whether the film would be woken up or fatigued was ~0.8 MV/cm, above or below which wake-up or fatigue was observed, respectively. Repeated cycling with sufficiently high voltages led to development of the interfacial nonpolar *P*4_2_/*nmc* phase, which induced fatigue through the depolarizing field effect. The fatigued film could be rejuvenated by applying a slightly higher voltage, indicating that these transitions were reversible. These mechanisms are radically different from those of conventional ferroelectrics.

## Abstract (web summary)

HfO_2_-based ferroelectric films are attracting a great deal of attention. Here, the authors conclude that the performance degradation and the possible rejuvenation are ascribed to the reversible transition between polar and antipolar phases.

## Introduction

Ensuring reliable and repeatable electrical performance from ferroelectric (FE) thin films and devices present a significant challenge for conventional FE materials such as perovskite-structured Pb(Zr,Ti)O_3_ (PZT) to enable their use in FE memory devices^1–4^. Among the potential problems, fatigue, which is induced by defect (mainly oxygen vacancy (V_O_)) generation^5^ and the accompanying FE domain pinning^6,7^ that occurs during repeated electrical stimulation, is one of the most critical concerns^1–4^. While the discovery of ferroelectricity in doped HfO_2_ thin films by Böske et al.^8^ in 2011 has again triggered enormous interest in FE memories^2–4^ and several other novel applications^1,9–11^, these films are not exempt from serious fatigue concerns^2,3^. However, the much higher binding energy of the Hf–O bond when compared with the Ti–O bond in PZT makes serious V_O_ involvement in the fatigue in this material unlikely.

Another interesting but also undesirable effect in this new FE material is wake-up (a temporal increase in the switchable polarization during the first 10^4^–10^5^ switching cycles)^12–16^. This has been ascribed to the decrease in the non-ferroelectric tetragonal interfacial layer (T-phase, *P*4_2_/*nmc*) caused by the reduced V_O_ concentration in the layer^13,15^. However, a reactive TiN electrode would be likely to increase the V_O_ concentration during repeated cycling^13,15^. Therefore, these two issues, i.e., severe fatigue and wake-up, as direct consequences of the V_O_ effect are hardly understood.

In fluorite-structured FE materials, four of the eight oxygen ions within each unit cell were shifted along the *c* axis direction (O_II_ ions), while the other four oxygen ions (O_I_ ions) remained in centrosymmetric positions^17,18^. The energy of the polar phase (FE *Pca*2_1_, where all O_II_ ions shift in the same direction) is only slightly higher than that of the antipolar phase (antiferroelectric (AFE) *Pbca*, where O_II_ ions shift into antiparallel directions) by ~10 meV/f.u^17,19^. These specific crystallographic features of HfO_2_-based FE materials may render the transition between the FE *Pca*2_1_ and AFE *Pbca* phases to be both fluent and reversible, depending on the bias voltage conditions, and maybe the origin of the fatigue and wake-up phenomena. However, this probability has scarcely been examined to date.

This work used an advanced imaging technique, Cs-corrected scanning transmission electron microscopy in annular bright-field mode (STEM-ABF), which enables reliable imaging of light atomic columns (oxygen) in the presence of much heavier atomic columns (Hf and Zr) in fluorite-structured (Hf_0.5_Zr_0.5_)O_2_ (HZO) films^20^. The pristine, woken-up, fatigued, and rejuvenated 10- to 15-nm-thick HZO films were prepared by electrical cycling of TiN/HZO/TiN FE capacitors. The atomistic structural evolutions occurring in each state were examined using the STEM-ABF technique. The involvement of V_O_ effect was observed during fatigue cycling, which increased the interfacial T-phase, but the direct cause of the fatigue differed fundamentally from that in conventional FE materials. The wake-up, fatigue, and rejuvenation were mediated by the (reversible) transition between the FE *Pca*2_1_ and AFE *Pbca* phases. This conclusion was further supported by theoretical calculations based on density functional theory (DFT). The theoretical findings on the switching path and the bias voltage dependence matched the experimental results very well.

## Results

A TiN/HZO/TiN capacitor with HZO thickness of 15 nm was fabricated using the atomic layer deposition (ALD) process at a substrate temperature of 280 °C (see “Methods” section). Because the FE orthorhombic phase (O_FE_-phase, *Pca*2_1_) is not the thermodynamically stable bulk phase^21,22^, it could be achieved by kinetic means, supplemented by the surface energy/stress effects in the fine-grained (~10 nm) polycrystalline thin films^17,23^. This phase formation could be enabled by a specific kinetic phase transition from the amorphous as-deposited ALD material during post-deposition annealing (PDA)^24–26^. The typical thickness of the HZO film with the highest remanent polarization (*P*_r_) was ~10 nm, but a slightly greater thickness (15 nm) was used in this work to ease the STEM characterization.

**Fig. 1:**
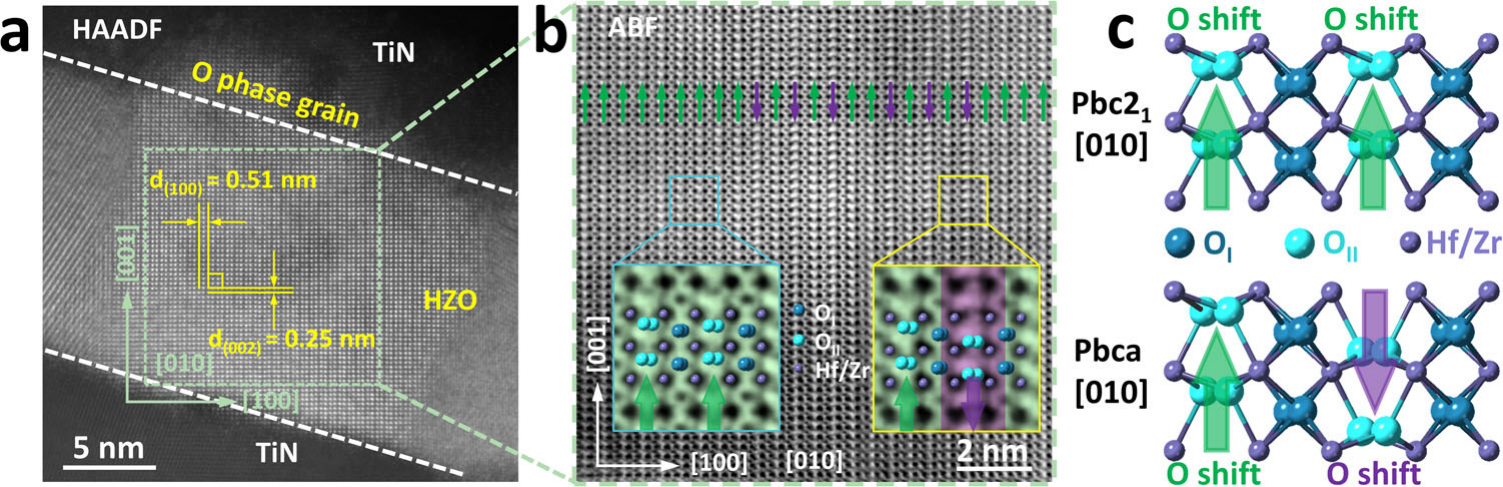
The observation of oxygen atoms of single orthorhombic (O-) phase grain in pristine TiN/Hf_0.5_Zr_0.5_O_2_ (HZO, 15 nm)/TiN device. **a** Scanning transmission electron microscopy (STEM) high-angle annular dark-field (HAADF) image of the TiN/HZO/TiN cross-section. **b** Annular bright-field (ABF) image extracted from the green square area in **a**, in which the position of the O atomic columns can be clearly detected with different offset and direction. According to the O atomic columns deviated from the center of the four nearest Hf/Zr columns, it can be divided into O_I_ type (center) and O_II_ type (off-center). Some of the O_II_-site columns shift along the [001], while others shift to the opposite direction. **c** The atomic models of the *Pbc*2_1_ and *Pbca* phases along [010] direction. The purple, navy blue, and cyan-colored solid balls correspond to the Hf/Zr, O_I_ (center), and O_II_ (off-center) ions, respectively.

Figure 1a shows the cross-section of the pristine (before electrical cycling) TiN/HZO/TiN structure, which was observed by STEM in the high-angle annular dark-field (HAADF) mode. In the figure, one of the grains in the HZO film that penetrates through the entire film thickness was identified. The bright contrast region corresponds to the Hf and Zr ionic columns, which confirmed that the grain’s crystal structure is O-phase. However, the different space groups (SGs) among the various O phases cannot be identified by recognition of heavy Hf/Zr ions alone. In particular, identification of the O_FE_-phase *Pca*2_1_ (SG: 29) and the antipolar O_AFE_-phase *Pbca* (SG: 61) is an advantage of the STEM-ABF technique, which has a high sensitivity to light elements, i.e., oxygen ions. Zooming in to the dashed green square area in Fig. 1a in STEM-ABF mode allowed the oxygen ion positions to be determined clearly, as shown in Fig. 1b. Using the shifts in the oxygen ion positions from the centers of the four nearest Hf/Zr ion positions, the oxygen ions can be divided into O_I_-type (navy blue, at the centrosymmetric position) and O_II_-type (cyan, at the off-center position). Here, for ease of comparison of the two crystal structures, the polar O_FE_-phase was identified as the *Pbc*2_1_ phase rather than the standard *Pca*2_1_, which causes the *a*-, *b*-, and *c* axis directions of the two O-phases to coincide. There are two shift directions for the O_II_-type ions: along the [001] direction (green arrows; majority of O_II_ ions) and along the $[00\bar{1}]$ direction (purple arrow; minority of O_II_ ions). Note that atomic-resolution TEM images show the projected positions of atoms along the beam direction (in this case, the [010] O-phase direction). If mixtures of O_I_ and O_II_ positions were present along this direction, there could hardly be a clear distinction between the O_I_ and O_II_ positions in the STEM-ABF image, but this was not the case (see the enlarged images in the insets). Therefore, there are two types of crystal region: one with all O_II_ ions shifting along the [001] direction (green arrows) and another with O_II_ ions shifting alternatingly along the [001] and $[00\bar{1}]$ directions (purple and green arrows, respectively), which can be identified more clearly in the enlarged insert images from Fig. 1b. The larger proportion with green arrows corresponds to the *Pbc*2_1_ O_FE_ domain with uniform polarization (*P*). The regions with alternating green- and purple-colored arrows might be regarded as the *Pbc*2_1_ O_FE_-phase with a one-unit-cell-thick FE domain width, but an even more appropriate interpretation would be that it is the O_AFE_ *Pbca* phase. Figure 1c exhibits the two kinds of atomic models of the *Pbc*2_1_ and *Pbca* phases along [010] direction, in which the purple, navy blue, and cyan-colored solid balls correspond to the Hf/Zr, O_I_ (center), and O_II_ (off-center) ions, respectively. A detailed atomic structure comparison of the O_FE_ *Pbc*2_1_ and O_AFE_ *Pbca* phases is shown in Supplementary Fig. S1, which contains self-consistent experimental and theoretical ABF images. These results indicate that many of the O-phase single grains in the pristine state are composed of a mixture of the O_FE_ *Pbc*2_1_ and O_AFE_ *Pbca* phases. This is expected because the free energies of these two phases are quite similar, and the crystallization stage during PDA processing thus involved a high probability of formation of these structures.

**Fig. 2:**
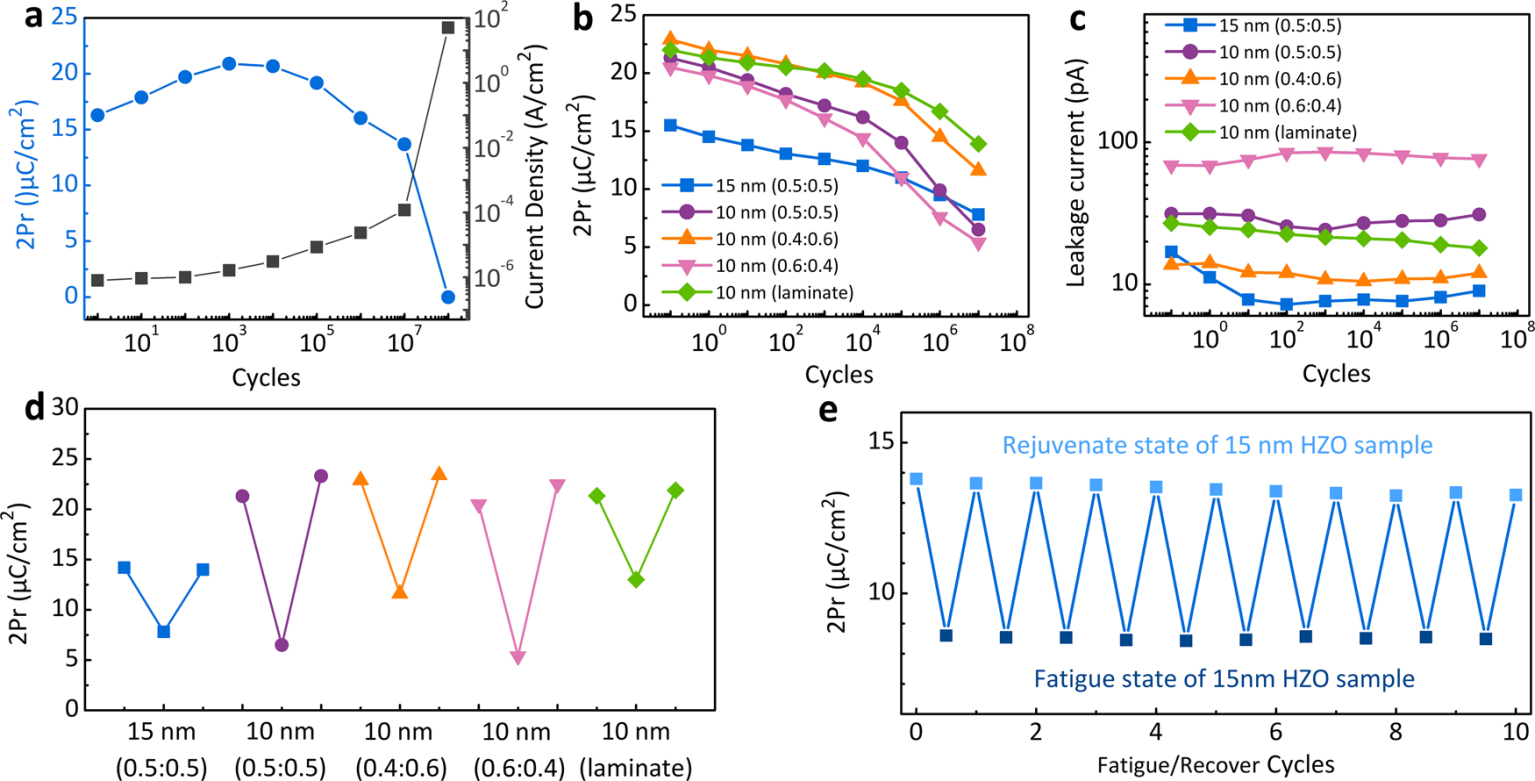
Electrical measurement results of different TiN/HZO/TiN devices: 15-nm-thick Hf_0.5_Zr_0.5_O_2_, 10-nm-thick Hf_0.5_Zr_0.5_O_2_, 10-nm-thick Hf_0.4_Zr_0.6_O_2_, 10-nm-thick Hf_0.6_Zr_0.4_O_2_, and the nanolaminated 10-nm-thick Hf_0.5_Zr_0.5_O_2_ films. **a** The 2*P*_*r*_ of 15-nm-thick Hf_0.5_Zr_0.5_O_2_ device measured by ±3 V *P–V* loop and the corresponding leakage currents measured at 2 V, as a function of switching cycles, which was performed by applying bipolar voltage pulses of ±5 V at f = 500 kHz. **b** The trend of 2*P*_*r*_ changes of different devices with the switching cycles at a low field of ±1.8 MV/cm. **c** The corresponding leakage current changes with the cycles. **d** The rejuvenation process of different devices by applying field pulses of ±3 MV/cm. **e** Ten cycles of fatigue/rejuvenation process on the 15-nm-thick Hf_0.5_Zr_0.5_O_2_ device under ±2.2 V, 5 × 10^6^ bipolar pulses for fatigue and ±4.0 V, 1 × 10^4^ bipolar pulses for recovery.

It is commonly believed that the hafnia-based ferroelectric capacitors undergo a wake-up-fatigue-breakdown trend under continuous switching cycles. Figure 2a shows the variations in 2*P*_r_ and the leakage currents of a TiN/Hf_0.5_Zr_0.5_O_2_ (15 nm)/TiN capacitor with increasing numbers of switching cycles (bipolar voltage pulses of ±5 V at *f* **=** 500 kHz). The 2*P*_r_ was measured by ±3 V *P–V* loop, and the leakage current was measured at 2 V. During the first 10^4^ cycles, wake-up occurs, and the leakage current remains almost constant. During subsequent cycling up to ~10^7^ cycles, the samples showed gradual fatigue (with 2*P*_r_ decreasing from ~20.7 to 13.7 μC/cm^2^) and a simultaneous increase in the leakage current by more than 50 times. The sample broke down completely after 10^8^ cycles. The field-dependent wake-up process was shown in Supplementary Fig. S2 (for the 15 nm Hf_0.5_Zr_0.5_O_2_ film), where all 2*P*_r_ values were measured using a ±3 V *P–V* loop after each decade of cycles. At low voltages (<3.2 V), no obvious wake-up was observed, but the 2*P*_r_ value decreased monotonically with increasing numbers of switching cycles. However, at *V*_a_ **>** 3.6 V, wake-up was clearly observed. Figure 2b shows the 2*P*_*r*_ evolution of samples with 15-nm-thick Hf_0.5_Zr_0.5_O_2_, 10-nm-thick Hf_0.6_Zr_0.4_O_2_, 10-nm-thick Hf_0.5_Zr_0.5_O_2_, 10-nm-thick Hf_0.4_Zr_0.6_O_2_, and the nanolaminated 10-nm-thick Hf_0.5_Zr_0.5_O_2_ films, under bipolar *V*_a_ pulses of ±1.8 MV/cm, which are lower than the critical wake-up field (Supplementary Fig. S3). The 2*P*_r_ values remained relatively constant up to ~10^4^ cycles and decreased rapidly toward much smaller values at 10^7^ cycles. The capacitor leakage currents were also measured at each decade of low-field cycling pulses, but no obvious increase in leakage current was observed, as shown in Fig. 2c. These findings indicated that the fatigue caused by low *V*_a_ values is irrelevant to the generation of new defects. Figure 2d shows the low-*V*_a_ fatigue and subsequent rejuvenation process. After the devices were fatigued using bipolar ±1.8 MV/cm, 10^7^ pulse cycles, and then rejuvenated by subsequent application of ±3 MV/cm, 10^4^ recovery pulse cycles. The rejuvenated capacitor could be fatigued and rejuvenated again, with ten cycles of repetitive fatigue/recovery process, as shown in Fig. 2e. This “temporary” low-*V*_a_ fatigue and rejuvenation phenomenon is quite different from the conventional perception of fatigue, which is generally regarded as an irrecoverable process that causes permanent *P*_r_ degradation and device failure^15,27,28^. These findings indicate that the changes in the internal (defect) structure of the HZO film during field cycling are different for the low and high *V*_a_ values. In the low-*V*_a_ cycling case, the main fatigue mechanism is probably the phase transition from the O_FE_ *Pbc*2_1_ to the O_AFE_ *Pbca* phase, while the rejuvenation can be ascribed to the reverse phase transition without involving permanent structural change or V_O_ generation. In contrast, the changes shown during high-*V*_a_ cycling may involve a specific permanent structural change, e.g., V_O_ generation and an accompanying phase transition. The subsequent STEM-ABF observations of the woken-up, fatigued, and rejuvenated samples confirmed the above hypotheses.

**Fig. 3:**
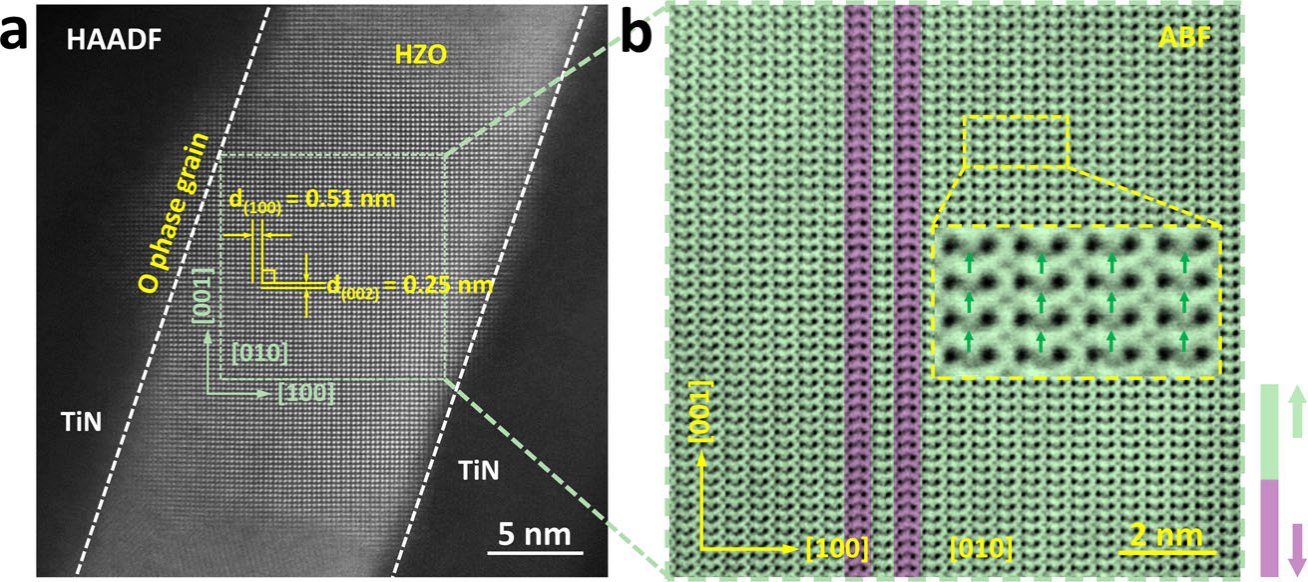
The observation of O_AFE_*Pbca* phase returning to O_FE_*Pbc*2_1_ phase after wake-up process. **a** Cross-section STEM-HAADF image of the TiN/HZO/TiN device after wake-up under the bipolar triangle pulses of ±4 V, 10^4^ cycles with f =500 kHz, in which a [010] oriented HZO O-phase grain is examined. **b** ABF image acquired from the green square area in **a**, the majority of the off-center O_II_ atomic columns shifted along the [001] direction (green), demonstrating a *Pbc*2_1_-dominated phase structure after the wake-up. The inset magnified ABF image shows the shifting direction of O_II_ atomic columns deviating from the center of the four nearest Hf/Zr columns, fitting well with *Pbc*2_1_ structure.

Figure 3a shows a STEM-HAADF image of the woken-up sample, which was cycled 10^4^ times at *V*_a_ = ±4 V and *f* = 500 kHz to wake-up the sample effectively but minimize the damaging effects of defect generation. The image shows an O_FE_- or O_AFE_-phase grain projected along the [010] zone axes. The corresponding STEM-ABF image is shown in Fig. 3b, in which the oxygen ion locations can be observed clearly. The image shows that most of the O_II_ atomic columns shift along the [001] direction (green), with only two vertical O_II_ columns with the opposite shift (purple) remaining; this indicates that almost the entire film becomes the O_FE_ *Pbc*2_1_ phase. The inset image in Fig. 3b shows a close-up that clearly reveals that all the O_II_ ions shifted in the same direction. Therefore, the wake-up effect could be correlated positively with the structural transition from the O_AFE_-phase in the pristine sample to the O_FE_-phase after cycling. This is a distinctive interpretation of the wake-up effect in this material when compared with the previous interpretation, which described it as the result of V_O_ redistribution from the interfacial nonpolar T-phase into the interior part of the O_FE_-phase film^15^. However, STEM observation of the interfacial region near the top electrode across the broad areas of both the pristine and woken-up samples (Supplementary Fig. S4a, b) showed that the interfacial T-phase thickness actually increased slightly, from 0.75–1.0 nm (pristine state) to 0.94–2.11 nm (woken-up state). This indicates that the interfacial nonpolar T-phase to O_FE_-phase transition may not be the major mechanism of the wake-up process. In contrast, the interpretation of the wake-up effect based on the O_AFE_-phase to O_FE_-phase transition in the bulk thin-film region does not involve such a difficulty. Additionally, it is consistent with the non-varying leakage current observed during the wake-up process (Supplementary Fig. S2).

**Fig. 4:**
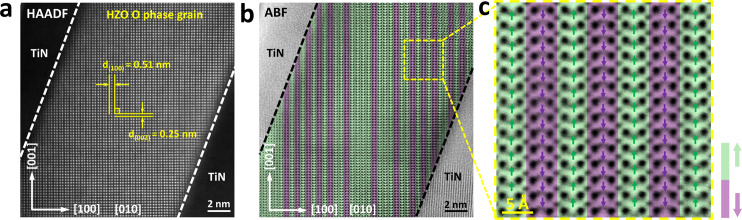
Phase transition from O_FE_*Pbc*2_1_ to O_AFE_*Pbca* phase after fatigue process. **a** Cross-section HAADF image of the TiN/HZO/TiN device after fatigue at V_a_ = ±2.5 V, f = 500 kHz, for 10^7^ times. The image shows an O-phase grain projected along [010] zone axis. **b** The corresponding ABF image of **a**, demonstrating clearly the off-center O_II_ atomic columns shifted along the [001] direction (green) and [00$\bar{1}$] direction (purple) alternately, showing a *Pbca* dominated structure. **c** The magnified ABF image acquires from the green square area in **b**, and the arrows show the deviating direction of O_II_ atomic columns from the center of the four nearest Hf/Zr columns.

The same reasoning implies that fatigue could also be induced by the reverse transition, i.e., from the O_FE_ *Pbc*2_1_ phase in the pristine state to the O_AFE_ *Pbca* phase after the fatigue. To confirm this hypothesis, a pristine 15-nm-thick Hf_0.5_Zr_0.5_O_2_ film was cycled 10^6^ times at *V*_a_ = ±2.5 V and *f* = 500 kHz, which corresponds to low-*V*_a_ fatigue without involving an intermediate wake-up. This cycling operation reduced the 2*P*_r_ value from ~9.4 to ~2.0 μC/cm^2^ (read at ±2.5 V, data not shown here). Figure 4a, b shows STEM-HAADF and STEM-ABF images of such a low-*V*_a_ fatigued sample, respectively. The images demonstrate that almost the entire area of the film was now in the O_AFE_-phase, which was further confirmed by the magnified view shown in Fig. 4c. To further verify the validity of the antipolar-polar transition model, the crystalline structure of other devices were examined at the low field fatigued and woken-up states, respectively. Supplementary Figs. S5–6 show the STEM-HAADF and STEM-ABF cross-section images of the 10-nm-thick nanolaminated Hf_0.5_Zr_0.5_O_2_ film. They also show a clear *Pbca* phase structure after the low-field fatigue and *Pbc*2_1_ phase-dominated structure after the wake-up. Similarly, Supplementary Figs. S7 and 8 show the reversible transition between the antipolar *Pbca* and polar *Pbc*2_1_ phases as the origin of the low-field fatigue and rejuvenation in the 10-nm-thick Hf_0.5_Zr_0.5_O_2_ film. As discussed below, the O_AFE_-phase free energy is lower than that of the O_FE_-phase by ~10 meV/f.u., thus indicating that this transition is energetically favorably driven. It was noted that the low-*V*_a_ fatigue only occurred with bipolar switching pulses. Stressing the device using unipolar voltages of the same amplitude did not cause fatigue (see Supplementary Fig. S9 and related discussion).

The question then becomes this: what is the critical field strength that determines whether fatigue or wake-up (and also rejuvenation) will occur? To answer this question, DFT was used to simulate the switching path from the *Pbca* global minimum energy phase to the *Pbc*2_1_ local minimum energy phase. Details of the calculations are included in SM (Supplementary Fig. S10). The effective electric field (*E*_eff_) distribution across the O_FE_ region of the film, which must be smaller than the applied field (*E*_app_) because of the presence of the non-FE layer, was also calculated by assuming the presence of the interfacial T-phase based on the STEM results (Supplementary Fig. S11). The presence of nonpolar layers in series with the FE film induces the depolarization field, which reduces the effective field greatly during the switching cycles. The goal of these calculations is to estimate the critical field (*E*_0_) that can make the polar O_FE_ phase more stable than the O_AFE_ phase by its electrostatic energy contribution.

**Fig. 5:**
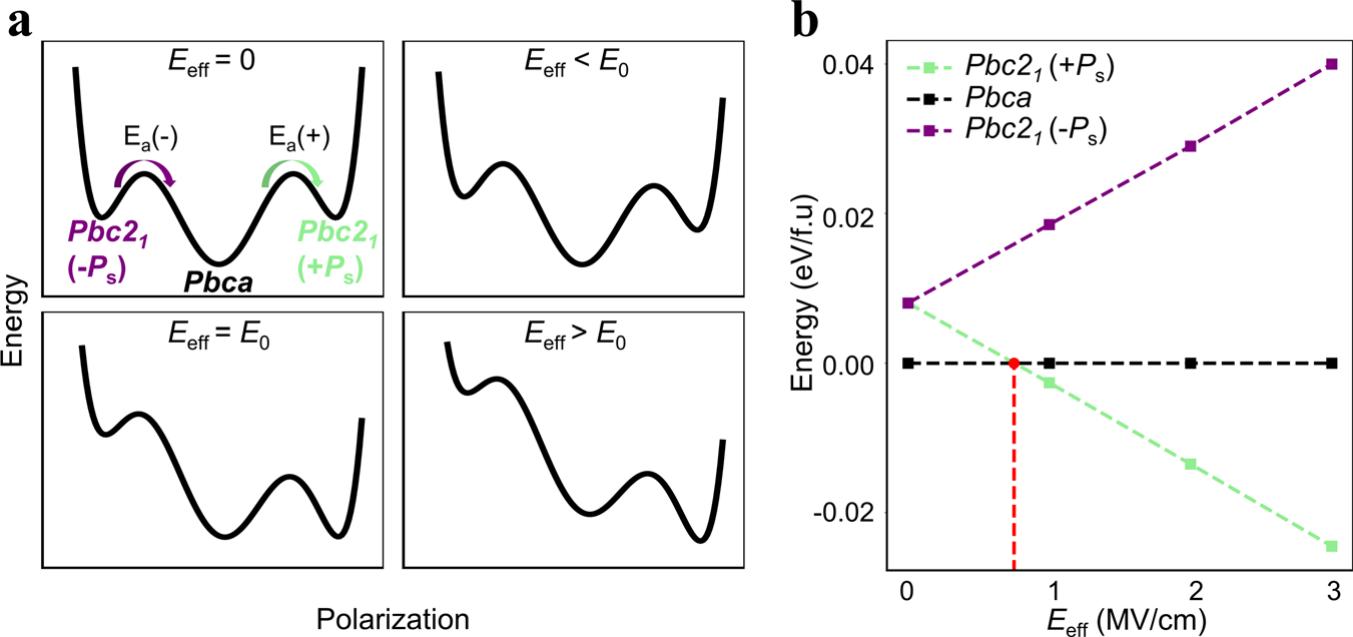
Energy landscape and DFT calculations. **a** schematic diagrams of the energy landscape for the *Pbca* (center global minimum) and *Pbc*2_1_ (two local minima at finite *P*) under zero *E*_eff_ and non-zero *E*_eff_. **b** calculated energies of the *Pbca*, *Pbc*2_1_(–*P*_s_), and *Pbc*2_1_(+*P*_s_) as a function of *E*_eff_.

Figure 5a shows the schematic diagrams of the energy landscape for *Pbca* (center global minimum) and *Pbc*2_1_ (two local minima at finite *P*). Under the application of a negative *E*_app_ (more precisely, *E*_eff_), the −*P*_s_ and +*P*_s_ (saturation polarization) states of the *Pbc*2_1_ phase become more unstable and stable, respectively, while the nonpolar phase energy remained unchanged. When *E*_eff_ > *E*_0_, the *Pbc*2_1_ phase becomes energetically more favorable than the *Pbca* phase; this is summarized in Fig. 5b. The calculations showed that *E*_0_ is ~0.8 MV/cm, i.e., when *E*_eff_ is lower or higher than 0.8 MV/cm, the O_AFE_ *Pbca* phase or the O_FE_ *Pbc*2_1_ phase becomes stable, respectively. The activation barrier (*E*_a_) of the direct transition between *Pbc*2_1_ and *Pbca* in HfO_2_ was also calculated, which is directly related to the wake-up, fatigue, and rejuvenation. They are summarized with the calculated relative energy of *Pbc*2_1_ to *Pbca* (ΔE), by ~10 meV/f.u., which is consistent with previous reports^17,19^ in Supplementary Table S1. Although the *E*_a_ depends on the transient path, i.e., the movement of O_II_ (off-center) atoms, these calculation results are comparable with previous studies, including the recent report by Xu et al. ^29^.

Table 1 shows *E*_eff_ across the O_FE_ layer when the interfacial T-phase layer is present within the 0.75–1.0 nm range in the pristine state and in the 0.94–2.11 nm range in the woken-up state (the first and second rows). When *V*_a_ is 3 V or higher, *E*_eff_ was higher than *E*_0_ for the pristine state, indicating that the film was prone to be woken-up, which is consistent with the results shown in Fig. 2c, except for the case where *V*_a_ is 3 V. This exception must be related to the still high activation barrier between the two phases (reaching as high as ~0.15 eV/f.u.), which prohibited fluent switching to the *Pbc*2_1_ phase under this condition. In the woken-up sample case, *E*_eff_ became smaller because of the involvement of the higher depolarizing effect. Therefore, a *V*_a_ of at least ~4 V was necessary to keep the *Pbc*2_1_ phase more stable than the *Pbca* phase during cycling. These calculation results strongly support the hypothesis for the wake-up and fatigue processes discussed in Figs. 1–4.

**Table 1:** Effective field strength (*E*_eff_) across the O_FE_ layer when the interfacial T-phase layer is present at *V*_a_ of 3, 4, and 5.5 V.

T-phase thickness (nm)	O_FE_-phase thickness (nm)	*E*_eff_ (MV/cm) at *V*_a_ = 3 V	*E*_eff_ (MV/cm) at *V*_a_ = 4 V	*E*_eff_ (MV/cm) at *V*_a_ = 5.5 V	Note
0.75~1.00	14.25~14.00	0.83~0.76	1.16~1.11	1.67~1.62	Pristine
0.94~2.11	14.06~12.89	0.78~0.49	1.12~0.84	1.63~1.37	Woken-up
3.25~4.25	11.75~10.75	0.19~−0.08	0.55~0.29	1.09~0.84	HF fatigue

Finally, the structural variations and accompanying electrical performances after fatigue cycling with a *V*_a_ of ±4 V are discussed. Supplementary Fig. S12a shows a STEM-HAADF image of the TiN/HZO/TiN capacitor after high-*V*_a_ fatigue testing (±4 V, 10^8^ cycles), which reduced the 2*P*_r_ value from 18.9 to 9.8 μC/cm^2^ (Supplementary Fig. S13a). The corresponding STEM-ABF image along the [010] direction in Supplementary Fig. S12b clearly shows the alternating distributions of the O_II_ ion positions being shifted along the [001] (green) and $[00\bar{1}]$ (purple) directions. This finding demonstrates that high-*V*_a_ fatigue was also induced by the transition from the O_FE_ *Pbc*2_1_ phase to the O_AFE_ *Pbca* phase. Another notable finding from this sample is the development of a fairly thick interfacial T-phase, which can be seen in Supplementary Fig. S12a. The STEM-HAADF images of this high-field fatigued sample confirmed that the thickness of this layer was ~3.25–4.25 nm. The last row of Table 1 indicates that *E*_eff_ across the remaining ~11.75–10.75 nm-thick O_FE_-phase is as low as 0.29–0.55 MV/cm for *V*_a_ = 4 V under these circumstances because of the severe depolarizing field effect. These calculation results thus corroborated the finding that high-*V*_a_ fatigue is induced by the transition from the *Pbc*2_1_ phase to the *Pbca* phase and not by defect generation and the accompanying domain pinning.

Following the same line of reasoning used for rejuvenation of the low-*V*_a_ fatigued sample shown in Fig. 2f, a similar rejuvenation was attempted using *V*_a_ = 5.5 V that increased *E*_eff_ to ~0.84–1.09 MV/cm, as also shown in Table 1. Supplementary Fig. S13b shows the evolution of the *P–V* curve with increasing rejuvenation cycling numbers up to 10^4^. As expected, the fatigued sample is partly recovered. This is further strong evidence that the high-*V*_a_ fatigue was also induced by the phase transition mentioned above.

Similar electrical and structural characterizations were performed for the 5.6-nm-thick HZO film to prove the general applicability of the reversible phase transition model for the thinner films. The results are summarized in Supplementary Figs. S14–S21. Although the thinner film contained a higher portion of T-phase grains in the pristine state, in addition to the O-phase grains, it showed similar wake-up, fatigue, and rejuvenation processes. Therefore, it can be inferred that the reversible transition between the nonpolar *Pbca* phase and polar *Pbc*2_1_ phase also occurs in the 5.6-nm-thick HZO film. The only difference is that the wake-up process in this thinner film is also contributed by the structural transition from the *P*4_2_*/nmc* T-phase to *Pca*2_1_ O-phase.

The knowledge acquired based on the effects of the V_O_ concentration on the phase stability indicates that the increase in the interfacial T-phase thickness is reasonable; it has been reported previously that the highest V_O_ concentration stabilizes the T-phase over the O- and monoclinic (M)-phases^28,30^. It must then be reasonable to believe that the repeated electrical stimuli enhanced the chemical interactions between the HZO film and the reactive TiN electrode, which would also increase the V_O_ concentration. It was also noted that these interfacial T-phases were mostly found around the top electrode interface. The bottom electrode interface was formed by depositing an HZO film on the TiN layer, which may produce a stable interface structure^31^. However, the top electrode interface was formed by depositing the TiN layer on the HZO film using a reactive sputtering process, which must have had a damaging effect on the HZO layer surface. Therefore, the top surface of the HZO layer must be vulnerable to oxygen concentration losses during electrical cycling tests.

## Conclusion

The crystal structures of pristine, woken-up, fatigued, and rejuvenated O-phase grains in atomic layer-deposited ferroelectric HZO films were examined in detail using the Cs-corrected STEM-HAADF and STEM-ABF techniques. The results showed that the 180° domain boundary structure of the O_FE_ *Pbc*2_1_ phase is identical to the crystal structure of the O_AFE_ *Pbca* phase, which is consistent with a previous theoretical expectation^18^. This structural relationship and the only slightly different energies of the two phases make the reversible phase change produced by the application of an electric field with a controlled magnitude feasible. The critical electric field for this transition was estimated to be ~0.8 MV/cm using DFT calculations.

The wake-up phenomenon could be induced by applying a voltage that exerted a switching field across the O_FE_ layer that was higher than the critical field. The interfacial T-phase grew as the number of switching cycles increased to the degree that was also dependent on the applied voltage. This interfacial T-phase generated a depolarizing field, which reduced the effective field applied to the remaining O_FE_ phase film. As the interfacial T-phase evolves in tandem with the increasing numbers of cycles, the effective field then becomes lower than the critical field, even at the given applied voltage; this is accompanied by the transition from wake-up to fatigue. However, because the fatigue in this material was not induced by irreversible defect generation and domain pinning but instead was induced by a (reversible) phase transition between the polar and antipolar phases, the fatigued sample could then be rejuvenated by applying a voltage that increased the effective field to exceed the critical field. This degradation mechanism for the ferroelectric performance of the HZO film is critically different from that of conventional perovskite-based FE thin films. A similar model can be applied to other fluorite-structured FE thin films, including variously doped HfO_2_ films.

While the oxygen loss caused by the repeated electrical stimuli was the fundamental origin of the performance degradation, it did not affect the phase stability and FE performance of the O_FE_ phase film directly. Instead, the evolution of the interfacial T-phase caused the depolarization field effect over the remaining O_FE_ phase-based portion, which then promoted the transition to the ground state O_AFE_ phase. Therefore, preventing oxygen loss during repeated electrical operations remains the most crucial factor in achieving the ultimate performance levels when using these newly established FE films.

## Methods

### Device fabrication

The TiN/HZO/TiN capacitor fabrication processes were as follows. First, the TiN bottom electrodes were deposited on the SiO_2_/Si substrate by ion beam sputtering. Then, 10–15-nm-thick HZO thin films were deposited by ALD at 280 °C. Hf[N(C_2_H_5_)CH_3_]_4_, Zr[N(C_2_H_5_)CH_3_]_4_, and H_2_O were used as the Hf precursor, the Zr precursor, and the oxygen source, respectively. The Hf:Zr ratio was controlled by alternating deposition of one cycle of HfO_2_ and one cycle of ZrO_2_. Also, 10-nm-thick nanolaminated HfO_2_/ZrO_2_ film, where each layer’s thickness was 1 nm, was prepared. Subsequently, the top electrode (TiN) layer was sputtered and patterned into different sizes. Finally, the as-fabricated TiN/HZO/TiN ferroelectric capacitors were annealed for 30 s at 500 °C in a nitrogen atmosphere to crystallize the HZO.

### Electrical measurements

The polarization–voltage (*P*–*V*) and leakage current characteristics of the metal-FE-metal devices were measured using a Radiant Workstation ferroelectric tester and an Agilent B1500 semiconductor parameter analyzer. During all electrical measurements, a bias voltage was applied to the bottom electrode, and the top electrode was grounded. The capacitor size was 100 µm × 100 µm.

### Cs-STEM experiment

After the different electrical operations, the TiN/HZO/TiN capacitors were processed into cross-sectional samples using the focused ion beam (FIB) technique in the FEI Helios G4 system, including low-pressure polishing processes at 5 and 2 keV. The sample was treated in a Gatan 691 precision ion polishing system at 1–0.5 keV to remove any residual contamination and damage from the sample surface. The STEM experiments were then conducted on a JEM Grand ARM 300F microscope operating at 300 kV in the STEM mode with a probe aberration corrector. The HAADF and ABF images were acquired at a probe convergence semi-angle of ~18 mrad. The STEM-HAADF images were acquired using an annular dark-field image detector with an inner semi-angle of more than 64 mrad. The STEM-ABF images were acquired using a bright-field image detector with its central section blocked using a beam stopper; the collection semi-angle was in the 12–24 mrad range.

### DFT calculations

Ab initio calculations were performed using the Vienna Ab-initio Simulation Package (VASP)^32,33^. The generalized gradient approximation of the Perdew–Burke–Ernzerhof (GGA-PBE) exchange-correlation functional^34^ and Blöchl’s projector augmented wave (PAW) approach^35,36^ were used with an energy cut-off of 600 eV. The *k*-points were sampled and found to be 8 × 8 × 8 for the *Pbc*2_1_ unit cell and 8 × 4 × 8 for the *Pbca* unit cell. The dielectric constant was obtained via density functional perturbation theory (DFPT)^37,38^, and the spontaneous polarization was calculated using the Berry phase method^39^. The activation barrier (*E*_a_) for switching was estimated using the solid-state nudged elastic band (SS-NEB) method^40^.

### Reporting summary

Further information on research design is available in the Nature Research Reporting Summary linked to this article.

## Supplementary information

Supplementary Information

Reporting Summary
