# Supplementary material for: Reversible transition between the polar and antipolar phases and its implications for wake-up and fatigue in HfO2-based ferroelectric thin film
Source: Nat Commun. 2022 Feb 3;13:645. doi: 10.1038/s41467-022-28236-5 (PMC8814215; doi:10.1038/s41467-022-28236-5)
Supplement: Supplementary file 1 — Supplementary Information [file 41467_2022_28236_MOESM1_ESM.pdf]

Supplementary Material

for

Reversible transition between the polar and antipolar phases in HfO_2-based ferroelectric thin film

Yan Cheng[1,#], Zhaomeng Gao[2,3,#], Kun Hee Ye[4,5], Hyeon Woo Park[4], Yonghui Zheng[1], Yunzhe Zheng[1], Jianfeng Gao[2], Min Hyuk Park[4,6], Jung-Hae Choi[5], Kan-Hao Xue[7], Cheol Seong Hwang[4*], and Hangbing Lyu[2,3*]

[1]Key Laboratory of Polar Materials and Devices (MOE), Department of Electronics, East China Normal University, 500 Dongchuan Road, Shanghai 200241, China

[2]Key Laboratory of Microelectronics Devices and Integrated Technology, Institute of Microelectronics, Chinese Academy of Sciences, No. 3 Bei-tu-cheng West Road, Chaoyang District, Beijing 100029, China

[3]University of Chinese Academy of Sciences, Beijing 100049, China

[4]Department of Materials Science and Engineering, and Inter-University Semiconductor Research Center, College of Engineering, Seoul National University, Seoul, 08826, Republic of Korea

[5]Electronic Materials Research Center, Korea Institute of Science and Technology, Seoul 02792, Republic of Korea

[6]School of Materials Science and Engineering, College of Engineering, Pusan National University, Busandaehak-ro 63beon-gil 2, Geumjeong-gu, Busan, 46241, Republic of Korea

[7]Wuhan National Laboratory for Optoelectronics, School of Optical and Electronic Information, Huazhong University of Science and Technology, Wuhan 430074, China

[#]Authors who contributed equally.

[*]Author to whom correspondence should be addressed: cheolsh@snu.ac.kr, lvhangbing@ime.ac.cn.

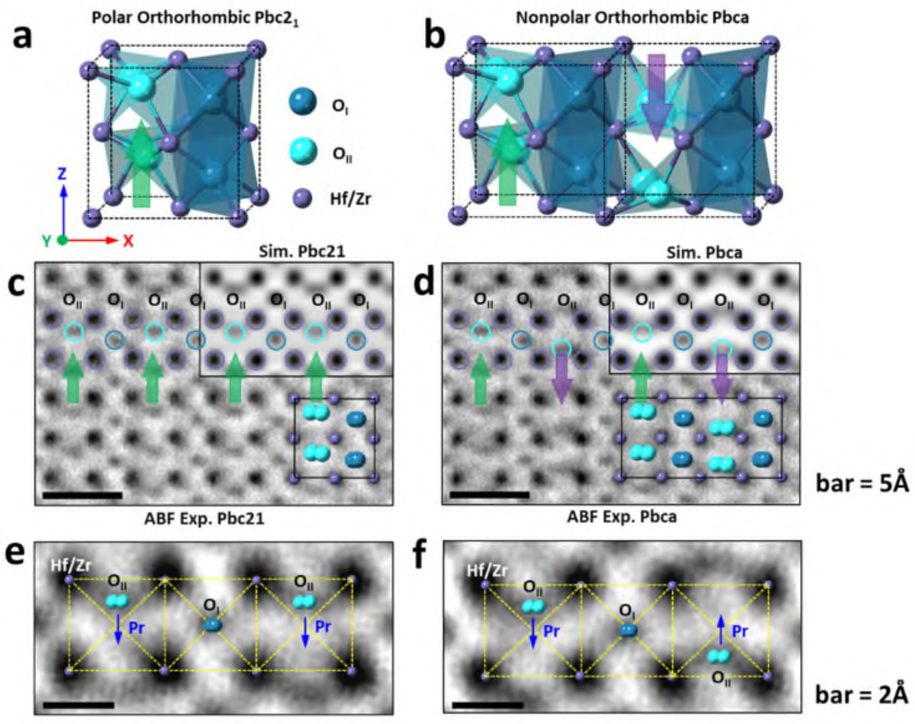

Figure S1. Atomic structures of the polar O_{FE}-*Pbc2₁* and nonpolar O_{AFE}-*Pbca* phases. The atomic structures of the (a) *$Pbc2_1$* and (b) *Pbca* phases in HZO obtained from density functional theory (DFT) calculations. The purple, navy blue, and cyan-coloured solid balls correspond to the Hf/Zr, O_I (centre), and O_{II} (off-centre) ions, respectively. (c) and (d) show atomic resolution annular brighT-field (ABF) images of HZO with insets containing the corresponding simulated ABF images along the [010] direction with space groups (SGs) of the *$Pbc2_1$* (29) and *Pbca* (61) phases. Scale bar = 5 Å. (e) and (f) show further magnified ABF images belonging to the polar *$Pbc2_1$* and nonpolar *Pbca* phases, in which the blue arrows indicate the direction of polarization. Scale bar = 2 Å.

The incoherent scattering of electrons, which is detected using the scanning transmission electron microscopy in the high-angle annular dark-field mode (STEM-HAADF) technique, is approximately proportional to Z^2, where Z is the atomic number. The atomic numbers of Hf, Zr, and Ti are 72, 40, and 22, respectively, and the HZO layer thus shows a brighter contrast amid the TiN/HZO/TiN structure. The STEM-HAADF imaging technique was incapable of providing the positions of the oxygen ions because of its low sensitivity to the lightweight element. The STEM-ABF technique has a high sensitivity to the oxygen ions and retains a Z-dependent image contrast

($\approx Z^{1/3}$), thus enabling reliable imaging of the light atomic columns (oxygen). In (c), the O_{II} ionic columns shift along the [001] direction of the unit cell defined by the four nearest Hf/Zr columns, which is consistent with the *Pbc2₁* phase. In (d), the O_{II} ionic columns shift along the [001] and $[00\bar{1}]$ directions alternately, which is strongly consistent with the *Pbca* lattice. The experimental ABF images match the simulated results well.

Method of STEM simulation: The software QSTEM[S1] with a multislice method was used to simulate the STEM-ABF images of *Pbc2₁* and *Pbca* phases. The microscope parameters are high voltage of 300 kV, convergence angle of 18 mrad, and ABF inner-to-outer angle of 12-24 mrad. The image simulation is performed along the [010] zone axes. A series of image simulations with different thicknesses, different sample angles, and different spherical aberrations were carried out to estimate their effect on the experimental results.

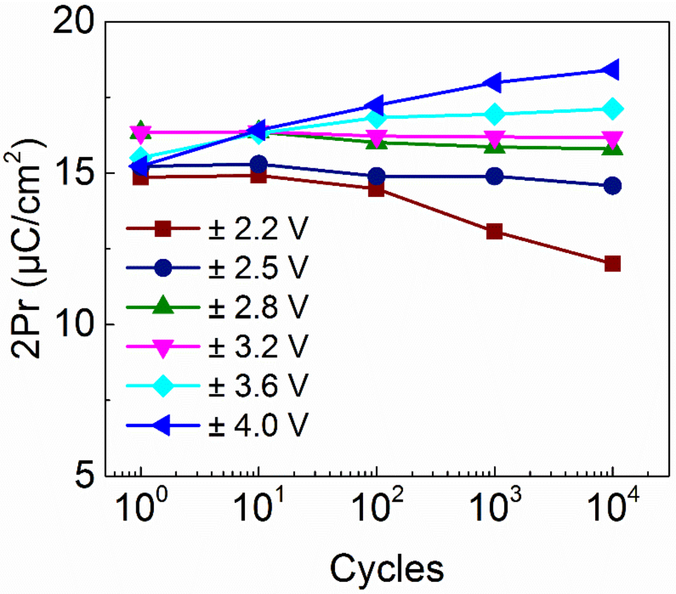

Figure S2. Field-dependent wakeup process of TiN/$Hf_{0.5}Zr_{0.5}O_2$(HZO, 15 nm)/TiN device. The device was stressed with the bipolar triangle voltage pulse with different pulse amplitudes of ±2.2 V, ±2.5 V, ±2.8 V, ±3.2 V, ±3.6 V, and ±4 V at f=500 kHz. The $2P_r$ value was read from the P-V curves with the voltage of ±3 V.

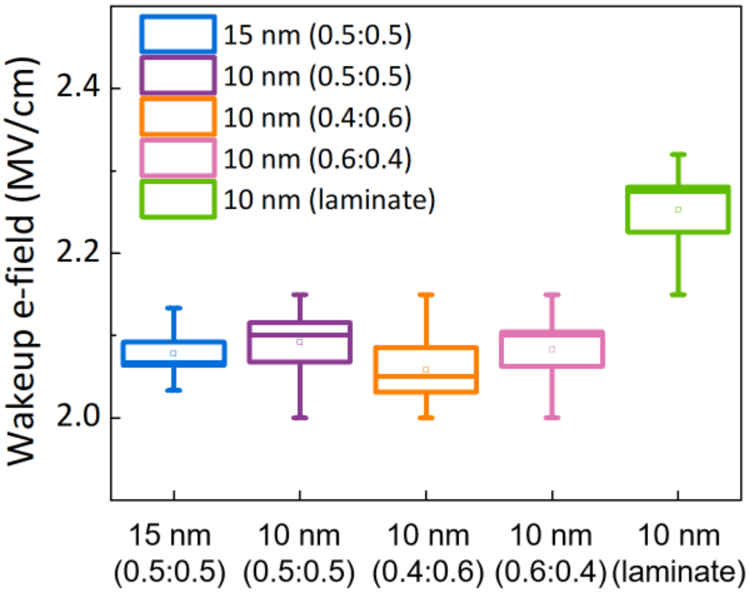

Figure S3. The wakeup electric fields of different TiN/HZO/TiN devices. 15 nm-thick $Hf_{0.5}Zr_{0.5}O_2$, 10 nm-thick $Hf_{0.5}Zr_{0.5}O_2$, 10 nm-thick $Hf_{0.4}Zr_{0.6}O_2$, 10 nm-thick $Hf_{0.6}Zr_{0.4}O_2$, and the nanolaminated 10 nm-thick $Hf_{0.5}Zr_{0.5}O_2$ films.

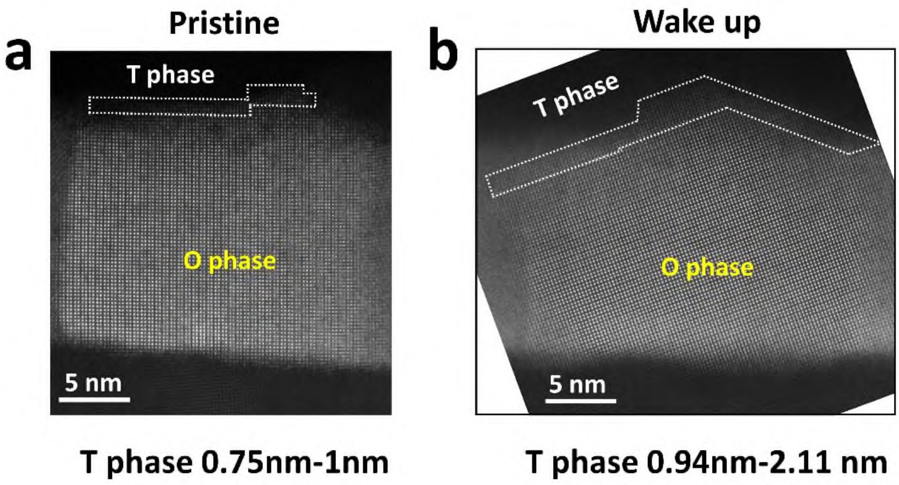

Figure S4. T-phase thickness measurements of the pristine and woken-up samples. (a) In the pristine sample, a thin T-phase layer was found at the top interface, with the layer thickness ranging from 0.75 nm to 1 nm. No T-phase layer was observed at the bottom interface. (b) In the woken-up sample, the upper T-phase layer thickness was found to increase slightly from 0.94 nm to 2.11 nm, but there was still no T-phase layer to be observed at the bottom interface.

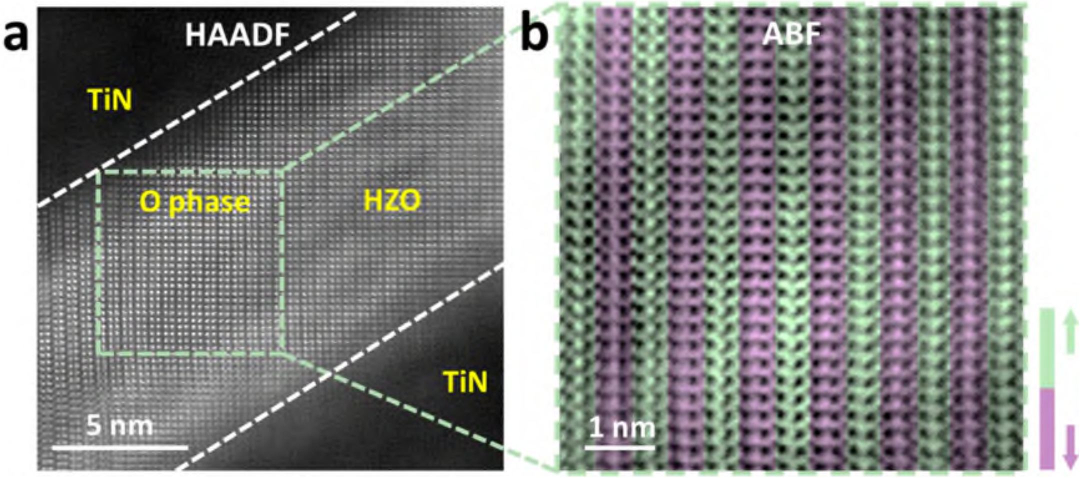

Figure S5. **The observation of O_{AFE} *Pbca* phase after the fatigue process.** (a) Cross-section HAADF image of the TiN/HZO/TiN device with the nanolaminated 10 nm-thick HZO film after fatigue at V_a= ±1.8 V, f=100 kHz, for 10^8 cycles. The image shows an O-phase grain projected along [010] zone axis. (b) ABF image acquired from the green square area in (a), demonstrating the off-center O_{II} atomic columns shifted along the [001] direction (green) and [00$\bar{1}$] direction (purple) alternately, showing a *Pbca* phase structure.

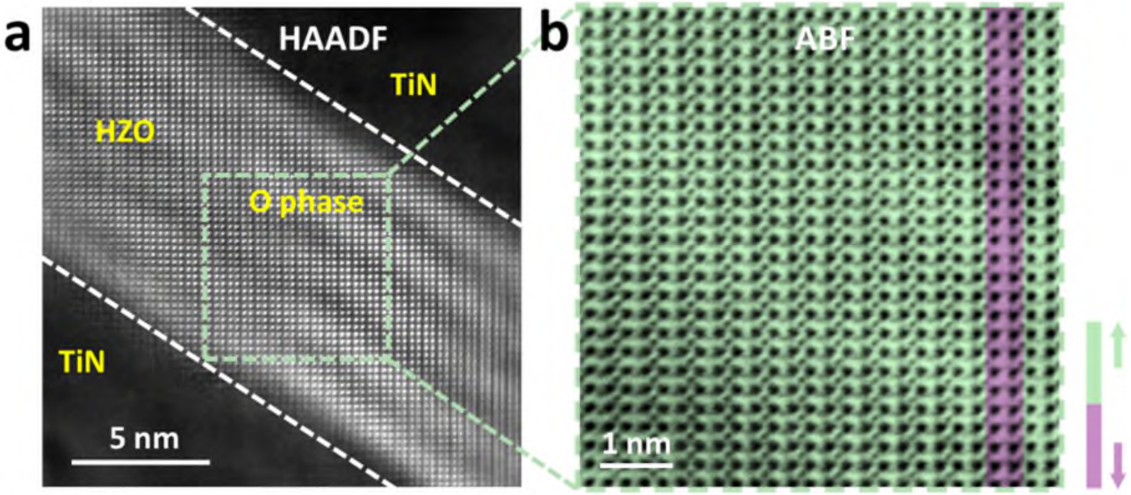

Figure S6. **The observation of O_{FE} *Pbc2₁* phase after the wakeup process.** (a) Cross-section STEM-HAADF image of the TiN/HZO/TiN device with the nanolaminated 10 nm-thick HZO film after wakeup under the bipolar triangle pulses of ±3.5 V, 10^4 cycles with f=100 kHz, in which a [010] oriented HZO O-phase grain is examined. (b) ABF image acquired from the green square area in (a), showing the majority of the off-center O_{II} atomic columns shifted along the [001] direction (green), and demonstrating a $Pbc2_1$-dominated phase structure.

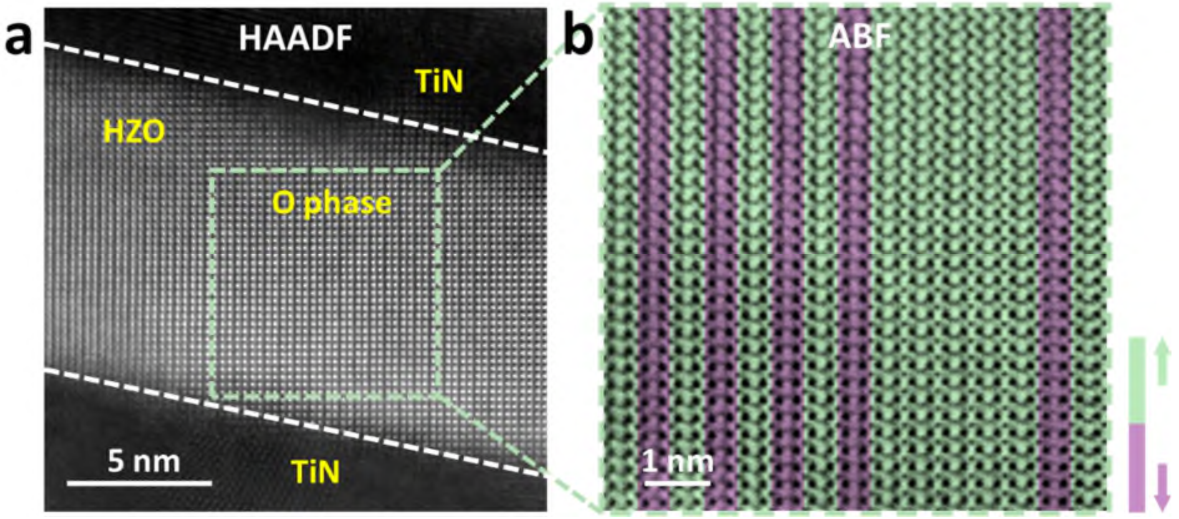

Figure S7. **The observation of O_{AFE} *Pbca* phase after the fatigue process.** (a) Cross-section STEM-HAADF image of the TiN/HZO/TiN device with the 10 nm-thick HZO film after fatigue under the bipolar triangle pulses of ±1.8 V, 10^7 cycles with f=100 kHz, in which a [010] oriented HZO O-phase grain is examined. (b) ABF image acquired from the green square area in (a), showing the off-center O_{II} atomic columns shifted along the [001] direction (green), and demonstrating a *Pbca* phase structure.

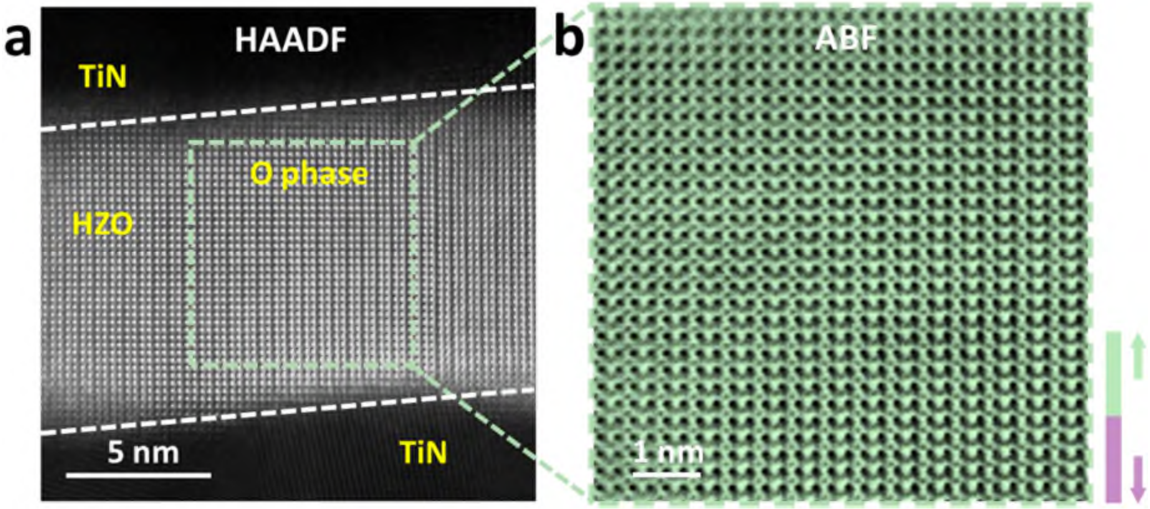

Figure S8. **The observation of O_{FE} *Pbc2₁* phase after the wakeup process.** (a) Cross-section HAADF image of the TiN/HZO/TiN device with the 10 nm-thick HZO film after wakeup at V_a= ±3 V, f=100 kHz, for 10^4 times. The image shows an O-phase grain projected along [010] zone axis. (b) ABF image acquired from the green square area in (a), demonstrating the majority of the off-center O_{II} atomic columns shifted along the [001] direction (green) and [00$\bar{1}$] direction (purple) alternately, showing a $Pbc2_1$ dominated phase structure.

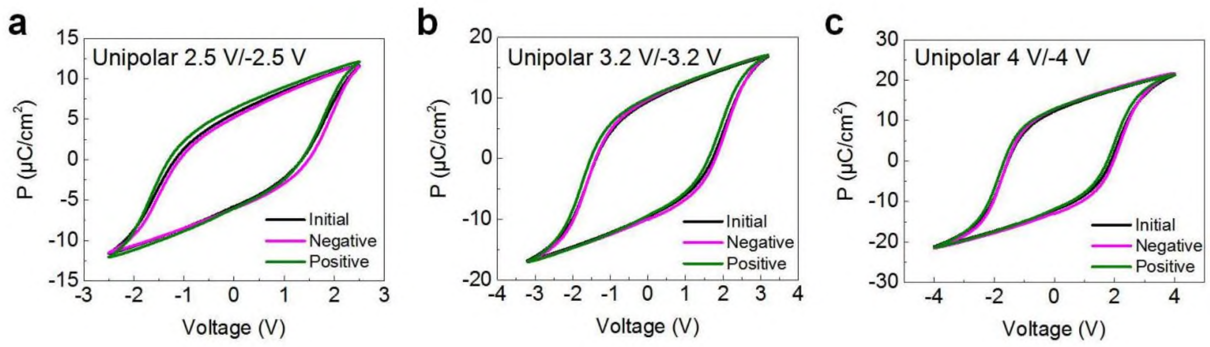

Figure S9. **Fatigue testing using unipolar pulses**. The device (15 nm HZO) was stressed using unipolar triangle pulses. (a) 2.5 V/-2.5 V; (b) 3.2 V/-3.2 V; (c) 4 V/-4 V. The tests showed that the unipolar pulses had little influence on fatigue. After 10^7 cycles with f=500 kHz, the device exhibited only a slight shift in V_c and showed almost no change in its $2P_r$ value, indicating that the application of unipolar pulses cannot lead to fatigue.

Stressing the device using a unipolar voltage with the same amplitude as the bipolar voltage did not lead to fatigue. This is a reasonable consequence of the FE polarization switching mechanism, which is mediated by the reverse domain nucleation and growth. During repeated application of bipolar pulses, the material continues to nucleate the reverse domains within the uniformly polarized FE domains. In conventional perovskites, this type of multi-domain configuration is generally less favourable than a uniform domain structure because of the involvement of the domain wall energy. However, the antiparallel domain wall configuration of the fluorite structure coincides with the crystal structure of the O_{AFE} *Pbca* phase, which has an energy that is lower than that of the O_{FE} $Pbc2_1$ phase. Therefore, it is highly feasible to have the O_{AFE} *Pbca* phase appear after repeated bipolar switching when the effective field was lower than the critical field, as mentioned in the main text. In contrast, unipolar electrical stressing could not induce such a structural change (i.e., multiple domain formation) because of the absence of reverse domain nucleation.

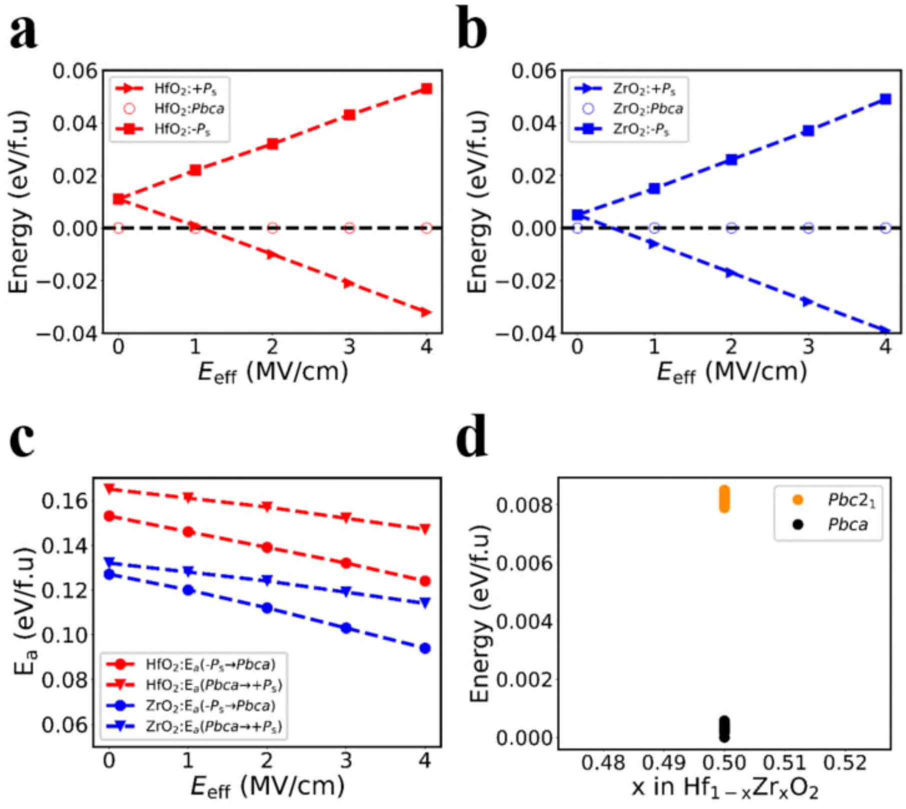

Figure S10. **DFT calculations**. Energies of (a) HfO_2 and (b) ZrO_2 as functions of E_{eff}. (c) Activation barriers from $Pbc2_1(-P_s)$ to *Pbca* and from *Pbca* to $Pbc2_1(+P_s)$ for HfO_2 and ZrO_2 as functions of E_{eff}. (d) Energies of $Hf_{0.5}Zr_{0.5}O_2$ in the $Pbc2_1$ and *Pbca* phases for more than 30 configurations for each phase.

The energy under the moderate-strength electrical field $E(E_{eff})$, was calculated, as shown in the literatures.[S2,S3]

$$\mathrm{E}(E_{eff}) = \mathrm{E_{DFT}} - V_0\, D\, E_{eff} = E_{\mathrm{DFT}} - \mathrm{V}_0\, (\varepsilon_r\, \varepsilon_o\, |E_{eff}|^2 + PE_{eff}),$$

where $\mathrm{E_{DFT}}$, V_0, D, ε_r, ε_o, P and E_{eff} are the energy calculated using DFT, the cell volume, the displacement, the relative dielectric constant, the dielectric constant of a vacuum, the spontaneous polarization, and the applied electric field, respectively. All values for $Hf_{0.5}Zr_{0.5}O_2$ in Fig. 5b are set as the arithmetic means of the corresponding values of HfO_2 and ZrO_2 based on the negligible dependence of the energy on the configuration in both the $Pbc2_1$ and *Pbca* phases, as illustrated in (d).

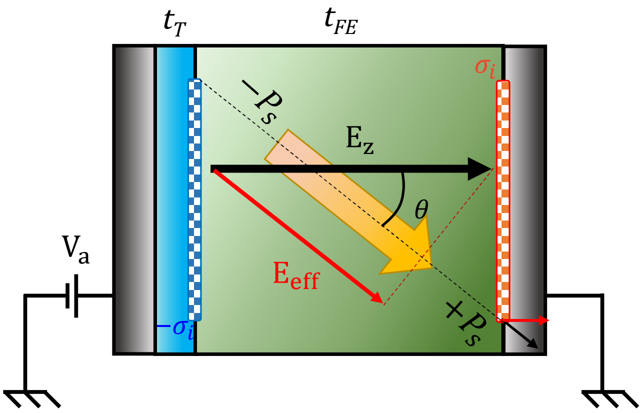

Figure S11. **Estimation of the critical field E_0**. E_0 was calculated when the energy of the polar O_{FE} phase became the same as that of the nonpolar O_{AFE} phase under the application of E_{eff}. The assumptions made here are: (i) the dielectric constant of the O_{FE} phase is isotropic; (ii) the orientations of the grains constituting the O_{FE} phase have a random distribution and thus the average of cosθ, denoted by $<\cos\theta>$, is 0.5; (iii) the depolarization field is fully uncompensated because of the highly insulating T-phase. Therefore, the screening coefficient is 1. The green and blue areas represent the O_{FE} phase and the T-phase, and the corresponding thicknesses are t_{FE} and t_T, respectively.

$$P_n = \vec{\boldsymbol{P}}_s \cdot \hat{\mathbf{n}}$$

$$E_z = \frac{C_T}{C_T + C_{FE}} \frac{V_a}{t_{FE}} - \frac{1}{t_{FE}} \frac{P_n + \sigma_i}{C_T + C_{FE}} = \frac{C_T}{C_T + C_{FE}} \frac{V_a}{t_{FE}} - \frac{1}{t_{FE}} \frac{\alpha P_n}{C_T + C_{FE}}$$

$$E_{eff} = E_z \cos\theta = \frac{C_T}{C_T + C_{FE}} \frac{V_a}{t_{FE}} \cos\theta - \frac{1}{t_{FE}} \frac{\alpha P_s}{C_T + C_{FE}} \cos^2\theta$$

$$< E_{eff} >= E_z < \cos\theta > = \frac{C_T}{C_T + C_{FE}} \frac{V_a}{t_{FE}} < \cos\theta > - \frac{1}{t_{FE}} \frac{\alpha P_s}{C_T + C_{FE}} < \cos^2\theta >$$

Here, E_{eff}, C_T, C_{FE}, V_a, σ_i, and P_s are the effective applied electric field, the capacitance of the T-phase, the capacitance of the O_{FE} phase, the applied voltage, the interfacial charge, and the spontaneous polarization, respectively.

Table S1. Calculated relative energy of *$Pbc2_1$* to *Pbca* (ΔE) and the calculated activation barrier from *Pbca* to *$Pbc2_1$* (E_a) in HfO_2 in comparison with the previous studies.

Transient path	ΔE [meV/f.u.]	E_a [meV/f.u.]	Pseudo potential	Cell relaxation	Ref.
local cubic	12	166	PBE	Y	This work
local Pbcm	12	111	PBE	Y	This work
NA	20	171	LDA	Y	[S4]
local Pbcm	12	117	PBE	NA	[S5]
local Pbcm	14	119	PBE	NA	[S6]
local monoclinic	13	138	PBE	Y	[S7]
Indirect transition*	18	110	LDA	Y	[S8]

NA; not available

* indirect transition (O_{AFE} ↔ T-phase ↔ O_{FE})

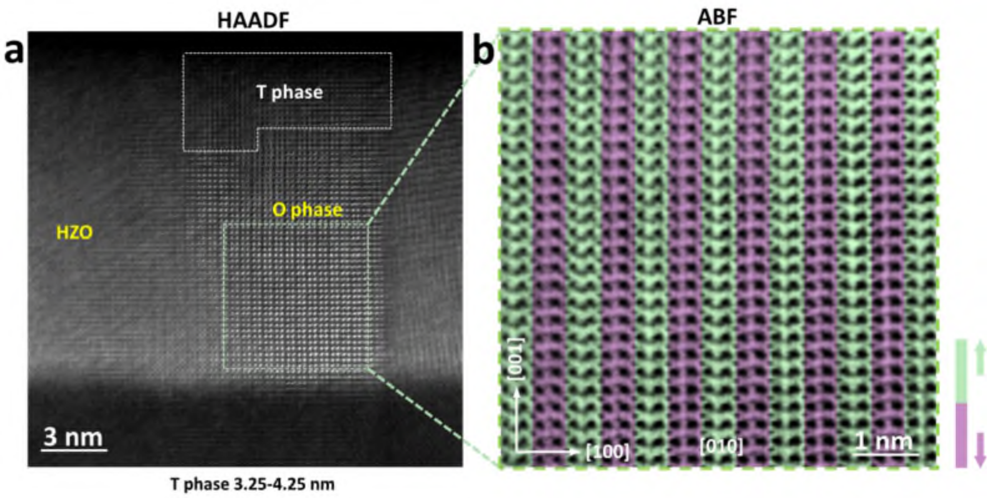

Figure S12. STEM characterization of the high-voltage fatigued sample. (a) STEM-HAADF image of the TiN/HZO(15 nm)/TiN capacitor after high-voltage fatigue testing (±4 V, 10^8 cycles). (b) Corresponding STEM-ABF image along the [010] direction. An alternating distribution of the O_{II} ion positions was observed, indicating that the high-voltage fatigue was also induced by the transition from the *Pbc2₁* to the *Pbca* phase with the development of a relatively thick interfacial T-phase, with thickness ranging from 3.25 nm to 4.25 nm.

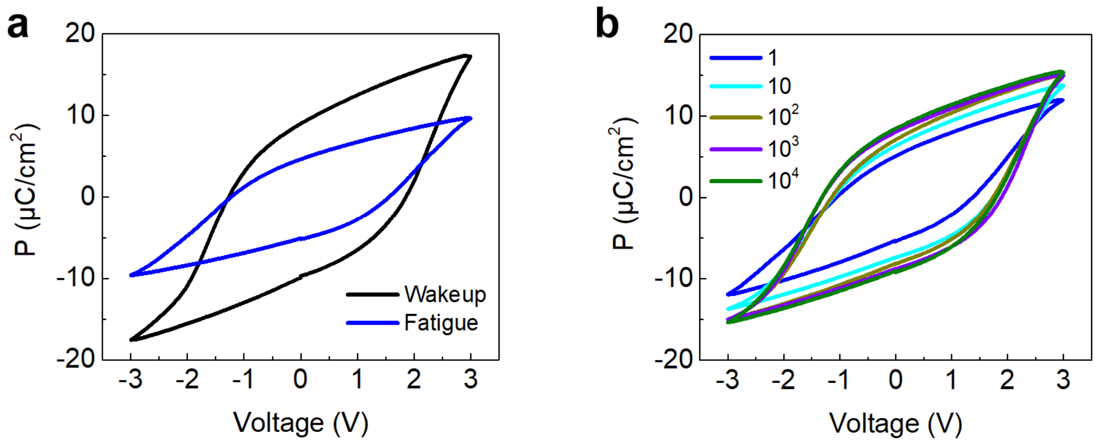

Figure S13. ***P*-*V* curves during the high-voltage fatigue and rejuvenation process**. (a) *P*-*V* curves of the initial state and the fatigued state with bipolar 10^8 cycling at ±4 V, *f*=500 kHz. $2P_r$ decreased from 18.9 μC/cm^2 to 9.8 μC/cm^2. The fatigue test was performed after wakeup. (b) *P*-*V* curves during the rejuvenation process. After the high-voltage fatigue process, the device was rejuvenated by applying bipolar ±5.5 V pulses with *f*=500 kHz. The *P*-*V* curves were recorded after each decade of pulses. The device could also be rejuvenated gradually, with $2P_r$ being recovered to 17.5 μC/cm^2 after 10^4 cycles. The partially recovered P_r could be ascribed to the thickened interface T-phase layer, matching well with the STEM observation shown in **Fig. S12a**.

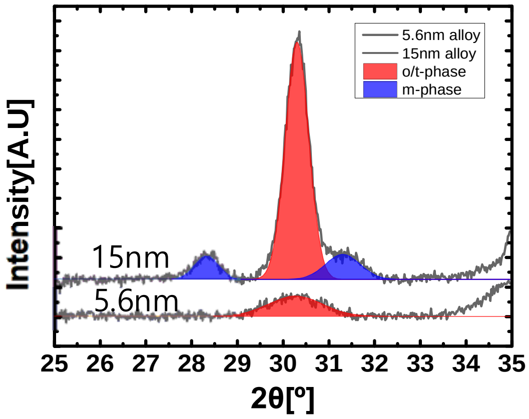

Figure S14. Grazing-angle incidence X-ray diffraction pattern of the 5.6 nm- and 15 nm-thick HZO film.

Figure S14 shows the GIXRD pattern of the pristine 15 nm- and 5.6 nm-thick films, showing that the 15 nm-thick film was composed primarily of O/T phase and minorly m-phase. In contrast, the 5.6 nm-thick film showed the negligible contribution of the m-phase, whereas the smaller grain size of this film broadened the diffraction peak shape substantially. This finding is consistent with previous reports. The broad peak shape rendered it improbable to identify whether the thinner film is mainly with O- or T-phase ($P4_2/nmc$). However, the following STEM data showed that both O- and T-phases coexisted.

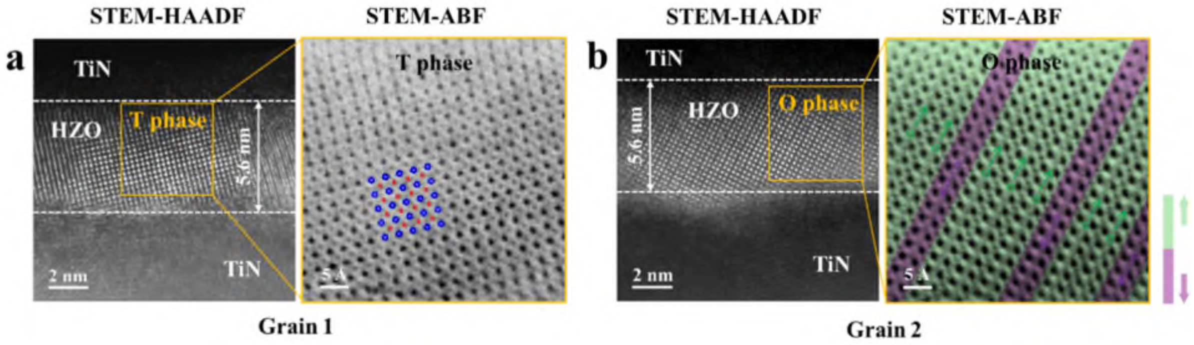

Figure S15. STEM-HAADF and STEM-ABF images of the pristine cross-section TiN/HZO (5.6 nm)/TiN device. (a) T-phase shows equidistant arrangement of Hf/Zr atoms and the centered oxygen atoms in the four nearest Hf/Zr atoms; (b) O-phase grains shows the coexistence of both the $Pbc2_1$ and *Pbca* structures.

Figures S15a and b show the representative STEM-HAADF/STEM-ABF images of the two grains with the T- and O-phase structures, respectively, of the 5.6 nm-thick film. Due to the significant difficulty of observing such well-identified grains with the favorable direction for the TEM observation in such thinner film, it was improbable to quantitatively state how high the relative portions of the two phases were. However, it was evident that both the T- and O-phases were included. They mostly have columnar shapes, i.e., both phase grains extended throughout the entire thickness without involving interfacial other phases, such as interfacial T-phase within the O-phase grain. The O-phase grain contained local structures corresponding to the $Pbc2_1$ (green arrows) and *Pbca* (purple arrows) phases. Therefore, it can be identified that the pristine 5.6 nm-thick HZO film was composed of T- and O-phase grains, where the O-phase grain included both the $Pbc2_1$ and *Pbca* regions.

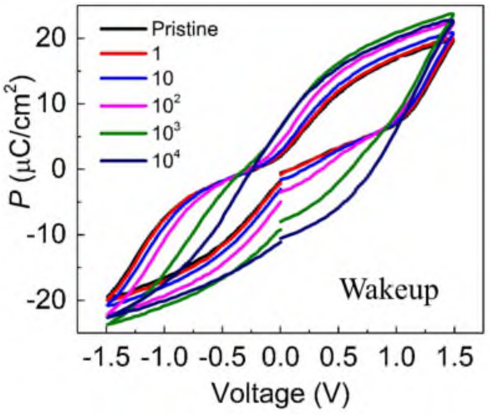

Figure S16. ***P-V*** **loops of TiN/HZO(5.6 nm)/TiN device during the wake-up process with bipolar ±4.4 MV/cm pulses.**

Figure S16 shows the evolution of the *P-V* curve of the 5.6 nm-thick film from the pristine state to the woken-up state after 1 x 10^4 cycles using the ±4.4 MV/cm pulses. The pristine sample showed a pinched anti-ferroelectric-like *P-V* loop, which is clearly different from the ferroelectric-like *P-V* loop of the 15 nm-thick film, shown in Figure S17. The pinched hysteresis loop is a representative feature of the T-phase hafnia-based film. As discussed by Zheng et al., [S9] such a wake-up process must be attributed to the change of the T-phase grains into O-phase by the electrical stress, as well as the transition of the *Pbca* phase to $Pbc2_1$ phase within the O-phase grain.

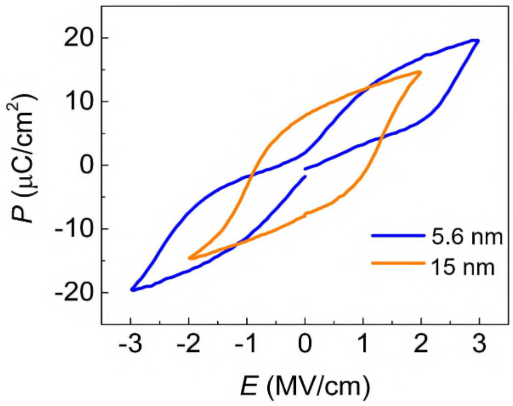

Figure S17. Pristine state *P-V* loops of TiN/$Hf_{0.5}Zr_{0.5}O_2$(5.6 nm)/TiN and TiN/$Hf_{0.5}Zr_{0.5}O_2$, (15 nm)/TiN samples.

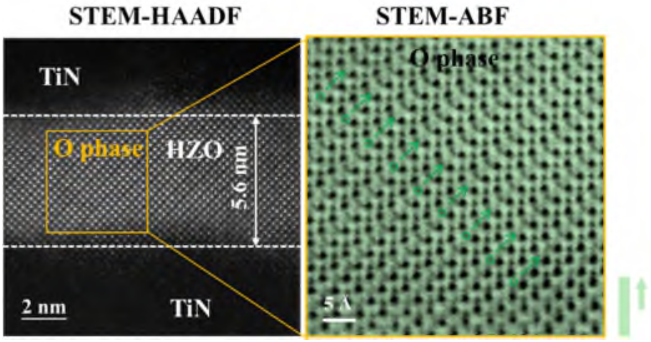

Figure S18. STEM-HAADF and STEM-ABF images of the woken-up cross-section TiN/HZO (5.6 nm)/TiN device, showing the uniformly transformed *Pbc2₁*-structure.

Figure S18 shows the STEM-HAADF and STEM-ABF images of the O-phase grain in the woken-up sample. They showed uniformly transformed $Pbc2_1$-phase grains with no evidence of the remaining *Pbca* phase. This finding indicates that the wake-up model developed for the 10-15 nm-thick film could also be applied to this thinner film, although the T-to-O-phase transition played a relatively minor role for the thicker films.

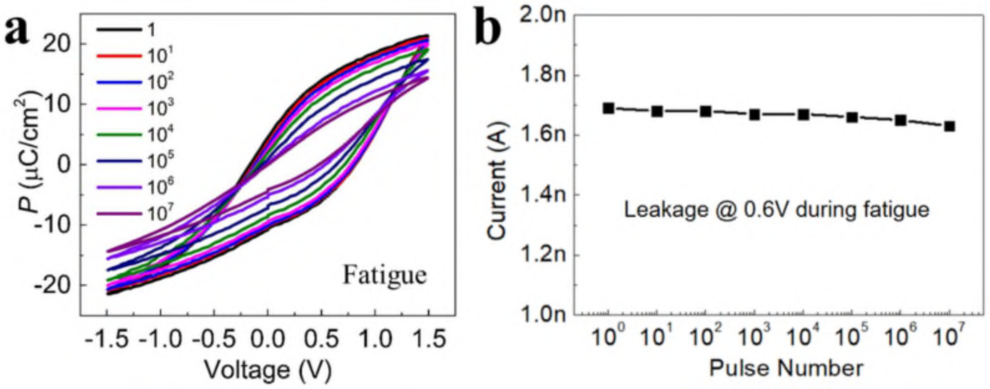

Figure S19. ***P-V*** **loops of TiN/HZO (5.6 nm)/TiN device during (a) low field fatigue process with bipolar ±2.0 MV/cm pulses, (b) Leakage current measurement during low field fatigue process at 0.6 V.**

After the wake-up, the film could be fatigued by using low field bipolar pulse (2 MV/cm), as shown in Fig. S19a. The leakage current during this low-field fatigue process keeps nearly constant (Fig. S19b). Unfortunately, achieving the STEM-HAADF and STEM-ABF images of the fatigued film was unsuccessful despite the extensive effort. Figure S20 shows a typical Cs-corrected HRTEM image of the fatigued film, showing many Móire fringes indicating the overlap of small grains along the electron beam direction. This condition is highly unfavorable to examine the oxygen ion locations.

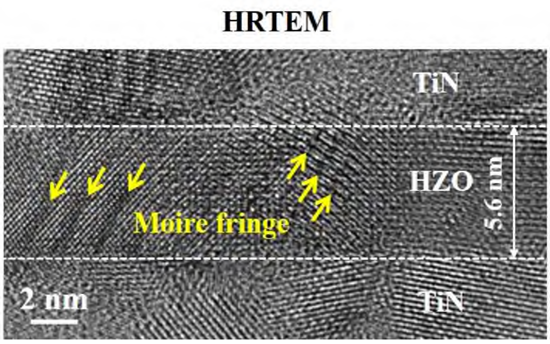

Figure S20. Cs-corrected HRTEM image of the fatigued film, showing many Móire fringes indicating the overlap of small grains along the electron beam direction.

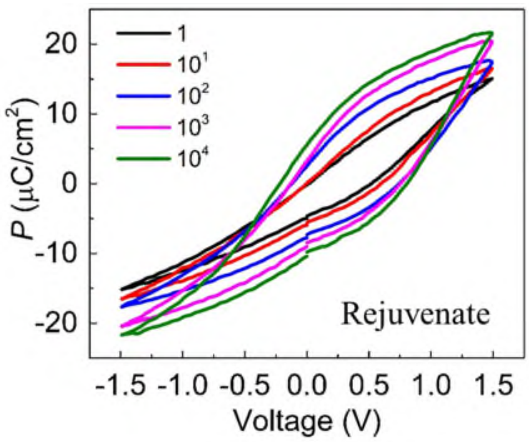

Figure S21. *P-V* loops of TiN/HZO(5.6 nm)/TiN device during rejuvenation process with bipolar ±4.4 MV/cm pulses.

After fatigue, it could also be rejuvenated by applying higher electrical pulses (±4.4 MV/cm, Fig. S21). This fatigue/rejuvenate phenomenon observed in the 5.6 nm-thick HZO film is identical to thicker films (10, 15 nm). The *P-V* loop of the 5.6 nm-thick HZO film after low-field fatigue did not show a pinched or anti-ferroelectric-like shape. Instead, it exhibits a paraelectric-like behavior. This finding suggested that the low field fatigue of the 5.6 nm-thick film does not result from the back-transition of its structure from the $Pca2_1$ O-phase to $P4_2/nmc$ T-phase. From the identical rejuvenation behavior of the fatigued thinner film to that of the thicker film, it can be inferred that the reversible transition between the nonpolar *Pbca* phase and polar $Pbc2_1$ phase also occurs in the 5.6 nm-thick HZO film. The only difference is that the wake-up process in this thinner film is also contributed by the structural transition from the $P4_2/nmc$ T-phase to $Pca2_1$ O-phase.

References

S1. Koch, C. T. Determination of Core Structure Periodicity and Point Defect Density Along Dislocations. Ph.D. thesis (Arizona State Univ., 2002).

S2. Nunes, R. W. & Gonze, X. Berry-phase treatment of the homogeneous electric field perturbation in insulators. *Phys. Rev. B* **63**, 155107 (2001).

S3. Souza, I., Íñiguez, J. & Vanderbilt, D. First-Principles Approach to Insulators in Finite Electric Fields. *Phys. Rev. Lett.* **89**, 117602 (2002).

S4. Liu, S. & Hanrahan, B. M. Effects of growth orientations and epitaxial strains on phase stability of HfO_2 thin films. *Phys. Rev. Mater.* **3**, 054404 (2019).

S5. Fan, S. T., Chen, Y. W. & Liu, C. W. Strain effect on the stability in ferroelectric HfO_2 simulated by first-principles calculations. *J. Phys. D. Appl. Phys.* **53**, (2020).

S6. Chen, Y. W., Fan, S. T. & Liu, C. W. Energy preference of uniform polarization switching for HfO_2 by first-principle study. *J. Phys. D. Appl. Phys.* **54**, 085304 (2021).

S7. Ding, W., Zhang, Y., Tao, L., Yang, Q. & Zhou, Y. The atomic-scale domain wall structure and motion in HfO_2-based ferroelectrics: A first-principle study. *Acta Mater.* **196**, 556–564 (2020).

S8. Xu, X. *et al.* Kinetically stabilized ferroelectricity in bulk single-crystalline HfO_2:Y. *Nat. Mater.* **20**, 826–832 (2021).

S9. Y. Zheng *et al.*, "In-situ atomic visualization of structural transformation in $Hf_{0.5}Zr_{0.5}O_2$ ferroelectric thin film: from nonpolar tetragonal phase to polar orthorhombic phase," *2021 Symposium on VLSI Technology*, 2021, pp. 1-2.
